# Hepatic oxylipin profiles in mouse models of Wilson disease: New insights into early hepatic manifestations

**DOI:** 10.1016/j.bbalip.2023.159446

**Published:** 2023-12-09

**Authors:** Tagreed A. Mazi, Noreene M. Shibata, Gaurav V. Sarode, Valentina Medici

**Affiliations:** a Department of Community Health Sciences-Clinical Nutrition, College of Applied Medical Sciences, King Saud University, P.O. Box 10219, Riyadh 11433, Saudi Arabia; b Department of Internal Medicine, Division of Gastroenterology and Hepatology, University of California Davis, 4150 V Street, Suite 3500, Sacramento, CA 95817, USA

**Keywords:** Wilson disease, Copper, Oxylipins, Reactive oxygen species, Inflammation, Cyclooxygenases, Lipoxygenases

## Abstract

Hepatic inflammation is commonly identified in Wilson disease (WD), a genetic disease of hepatic and brain copper accumulation. Copper accumulation is associated with increased oxidative stress and reactive oxygen species generation which may result in non-enzymatic oxidation of membrane-bound polyunsaturated fatty acids (PUFA). PUFA can be oxidized enzymatically via lipoxygenases (LOX), cyclooxygenases (COX), and cytochrome P450 monooxygenases (CYP). Products of PUFA oxidation are collectively known as oxylipins (OXL) and are bioactive lipids that modulate hepatic inflammation. We examined hepatic OXL profiles at early stages of WD in two mouse models, the toxic milk mouse from The Jackson Laboratory (tx-j) and the *Atp7b* knockout on a C57Bl/6 background (*Atp7b*^−/−^ B6). Targeted lipidomic analysis performed by ultra-high-performance liquid chromatography-electrospray ionization-tandem mass spectrometry showed that in both tx-j and *Atp7b*^−/−^ B6 mice, hepatic OXL profiles were altered with higher thromboxane and prostaglandins levels. The levels of oxidative stress marker, 9-HETE were increased more markedly in tx-j mice. However, both genotypes showed upregulated transcript levels of many genes related to oxidative stress and inflammation. Both genotypes showed higher prostaglandins, thromboxin along with higher PUFA-derived alcohols, diols, and ketones with altered epoxides; the expression of *Alox5* was upregulated and many CYP-related genes were dysregulated. Pathway analyses show dysregulation in arachidonic acid and linoleic acid metabolism characterizes mice with WD. Our findings indicate alterations in hepatic PUFA metabolism in early-stage WD and suggest the upregulation of both, non-enzymatic ROS-dependent and enzymatic PUFA oxidation, which could have implications for hepatic manifestations in WD and represent potential targets for future therapies.

## 1. Introduction

Wilson disease (WD) is an autosomal recessive disorder caused by a loss of function mutations in the *ATP7B* gene, which encodes for the transmembrane copper-transporter, P-type ATPase beta [1]. These mutations impair biliary copper excretion and result in excessive copper accumulation in the liver and other tissues [2]. WD age of onset is broad and clinical presentations are variable, including hepatic and neurologic/psychiatric signs and symptoms with abnormalities shown also in kidneys and heart [2]. The hepatic manifestations of WD include hepatocellular steatosis with various degrees of necro-inflammation, cirrhosis, and fibrosis that may progress to liver dysfunction and failure [3]. WD is also characterized by dysregulation in lipid metabolism, with impaired cholesterol synthesis, altered lipogenesis and plasma lipidomic profile [4–6]. If left untreated, WD can be disabling and fatal due to its multi-organ manifestations.

The toxic milk mice from The Jackson Laboratory (tx-j) and the *Atp7b* global knockout on a C57Bl/6 background (*Atp7b*^−/−^ B6) are two rodent models of WD. The tx-j mouse is characterized by a spontaneous missense mutation of the *Atp7b* gene, while the *Atp7b*^−/−^ B6 mouse is genetically engineered with global inactivation of the same gene and was originally generated to define the role of the genetic background in comparison with the *Atp7b*^−/−^ hybrid mouse model [7,8]. Similar to human WD phenotype, both mouse models show features of copper accumulation and hepatic manifestation and are considered valid models to investigate the pathological mechanisms of liver injury in WD. The natural history of liver pathology in tx-j mice is characterized by early fibrosis by 24–28 weeks of age, preceded by inflammatory infiltrate starting at 20 weeks with hepatocyte irregular and enlarged nuclei. The *Atp7b*^−/−^ B6 mouse model is particularly well characterized and it shows remarkable liver regeneration features with hyperplastic nodules as observed in the *Atp7b*^−/−^ model with hybrid phenotype [9] as well as marked inflammation with mononuclear hepatitis. Therefore, the *Atp7b*^−/−^ B6 model can be used to study compensatory regulatory mechanisms and metabolic changes in the state of liver disease related to copper toxicity [7,8,10]. At 16 weeks of age, we have also demonstrated extensive metabolic derangements associated with gut microbiome differences in both mouse strains. Therefore, both mouse models are very predictable in terms of copper accumulation and chronic liver damage with fibrosis development and presence of regenerative features.

The mechanisms by which copper induces tissue toxicity are not well understood. In WD, oxidative stress is a hallmark feature that intensifies with disease progression, and is thought to play a role in its pathogenesis [11]. When free/aqueous copper ions are present, these are thought to induce the formation of reactive oxygen species (ROS), as hydroperoxyl and hydroxyl radicals, via Fenton- and Haber-Weiss reactions [12], which may form adducts with proteins and DNA bases and impair cellular structures and machineries [13,14]. However, in WD, these irreversible changes are associated with substantially augmented hepatocellular oxidative stress and are features of advanced stages of liver disease, rather than early stages [11,15]. To date, the early drivers of hepatocellular damage in WD are not clear. Most of the available studies investigated liver samples with advanced liver damage, and the limited work available examining stages associated with minimal liver changes indicate that oxidative stress and lipid peroxidation markers are unaffected, however increasing with the progression of liver damage [16–18].

Also, ROS may react with membrane-bound unsaturated fatty acids and result in lipid peroxidation. Polyunsaturated fatty acids (PUFAs) may undergo ROS-dependent non-enzymatic auto-oxidation at a rate that is inversely correlated with oxidative stress to generate lipid mediators including 9-hydroxyeicosatetraenoic acid (9-HETE) which is derived from arachidonic acid (C20:4n6, AA) and considered an in vivo marker of oxidative stress, and 9-hydroxyeicosapentaenoic acid (9-HEPE) which is derived from eicosapentaenoic acid (C20:5n3) [19,20]. Alternatively, PUFAs may undergo enzymatic oxygenation in reactions catalyzed by cyclooxygenases (i.e. COX-1 and −2) to produce lipid mediators including prostaglandins (PGs) and thromboxane B_2_ (TXB_2_); lipoxygenases (e.g. 5-, 12-, and 15-LOXs) to produce fatty acid alcohols, ketones, hydroperoxides, lipoxins, and resolvins; and members of the cytochrome P450 monooxygenases (CYPs) to produce fatty acid alcohols and epoxides, and the latter are further hydrolyzed by the action of soluble epoxide hydrolases (sEH) to vicinal diols [21,22]. Products of PUFA oxidation are bioactive signaling lipids, collectively known as oxylipins (OXL). This diverse class acts in an autocrine, paracrine, or endocrine fashion via receptors to mediate processes, including lipid metabolism and inflammation, and are recognized components of the hepatic inflammatory milieu in multiple liver diseases [21–27]. For example, lipidomic analyses showed that dysregulated PUFAs metabolism and altered OXLs profile is associated with metabolic dysfunction-associated steatotic liver disease (MAFLD) and can predict its severity [28–31]. However, lipidomic profiling studies are limited in WD and the hepatic tissue lipidome is uncharacterized [5,32]. It is not clear if early stages of liver disease in WD are associated with alterations in the hepatic OXL profile that is relevant to inflammation and oxidative stress. This knowledge is important as it will shed light on pathways implicated in the onset and progression of liver disease in WD and highlight potential early therapeutic targets aimed at its delay, amelioration, or prevention.

To fill in current knowledge gaps, the objective of this study is to examine hepatic OXL profiles in two mouse models of WD, tx-j and *Atp7b*^−/−^ B6 mice, compared with corresponding controls at an early stage of hepatic disease. To accomplish this, we conducted quantitative lipidomic profiling of OXLs in liver tissue performed by ultra-high-performance liquid chromatography-electrospray ionization-tandem mass spectrometry (UPLC-ESI-MS/MS).

## 2. Animals and methods

### 2.1. Mouse models and diets

The *Atp7b*^−/−^ B6 mouse was generated as previously described [33] and provided by Dr. Svetlana Lutsenko at Johns Hopkins University. C3He-*Atp7b*^*tx*-*J*^/J toxic milk mice (tx-j, #001576) and C3HeB/FeJ control mice (C3H, #000658) were purchased from the Jackson Laboratory. All three strains were bred and maintained on the UC Davis campus under the following conditions: 20–23 °C, 40 %–65 % relative humidity, 14 h light/10 h dark light-cycle, and ad libitum LabDiet 5001 (Cu 13 ppm; Fe 240 ppm; Zn 75 ppm) (PMI, St. Louis, MO) and deionized water. Mice were housed 2–4 per cage. Tx-j and C3H control were maintained as homozygous colonies. Homozygous tx-j dams cannot support proper growth and development of their pups due to copper deficiency in their milk; therefore, tx-j pups must be fostered to a dam with normal milk to avoid death from copper deficiency within 14 days post-partum. C3H dams in the same phase of lactation as the biological tx-j dams (3–5 days postpartum) were used as foster dams, with tx-j pups raised alongside the C3H dam’s biological pups. The *Atp7b*^−/−^ B6 colony was maintained by heterozygous breeding and genotyped in-house by standard PCR.

At 16 weeks of age, *Atp7b*^−/−^ B6 (*n* = 23) and their *Atp7b*^+/+^ controls (WT) (*n* = 25), and tx-j mice (*n* = 18) and C3H controls (*n* = 12), were weighed and then anesthetized with isoflurane, exsanguinated retro-orbitally, euthanized by cervical dislocation, and their livers dissected and weighed. The exsanguination procedure implies removal of almost all the mouse blood (from 750 μL to a maximum of 1 mL), therefore livers do not require perfusion. All livers were sectioned into portions for flash-freezing in liquid nitrogen and for fixation in 10 % formalin. Blood was centrifuged at 6000 ×*g* for 10 min and plasma aliquoted. All samples were stored at −80 °C until further analysis. All protocols were approved by the UC Davis Institutional Animal Care and Use Committee and follow the National Research Council’s Guide for the Care and Use of Laboratory Animals.

### 2.2. Hepatic copper content

Fifty milligrams liver was digested in a 4:1 concentrated nitric acid to hydrogen peroxide matrix then diluted and analyzed on an iCAP Q Inductively Coupled Plasma Mass Spectrometer (ThermoFisher Scientific, Waltham, MA). All samples were processed and analyzed by the Northwestern University Quantitative Bio-element Imaging Center.

### 2.3. Histology

Formalin-fixed liver was transferred to 70 % ethanol then paraffin-embedded, sectioned, stained with hematoxylin and eosin, and whole-slide images scanned by the UC Davis Center for Genomic Pathology Laboratory.

### 2.4. Targeted lipidomic analysis

Liver samples were quantitatively profiled for non-esterified OXLs by ultra-high-performance liquid chromatography-electrospray ionization-tandem mass spectrometry (UPLC-MS/MS). Liver tissue extraction was performed as described [34]. Briefly, liver samples kept on dry ice were treated with antioxidant solution (0.2 mg/mL solution butylated hydroxytoluene (BHT)/ethylenediaminetetraacetic acid (EDTA) in 1:1 (methanol:water)) enriched with 10 μL of analytical deuterated surrogates, and 500 uL of cold methanol was added as an extraction solvent. Samples were homogenized, centrifuged, and subsequently evaporated to dryness in 50 nM of 1-phenyl 3-hexadecanoic acid urea (PHAU)/internal standards or 1-cyclohexyl-dodecanoic acid urea (CUDA) in methanol/acetonitrile (ACN) 50:50 and stored at −20 °C until analysis. Analytes were detected using API 4000 QTrap (AB Sciex, Framingham, MA) with a Thermo Scientific^™^ Vanquish^™^ UHPLC. This analytical platform detected 68 lipid mediators including fatty acid alcohols, ketones, epoxides, vicinal diols, PGs and TXB, and nitro lipids. The analysis was performed at the UC Davis West Coast Metabolomics Center. Details on the study and analysis protocol is available on the Metabolomics Workbench (http://www.metabolomicsworkbench.org), study ID number (ST002800). In this manuscript, abbreviations used for OXLs follow standard consensus. Details on lipid names, classes, and identifiers are shown in Table S1.

### 2.5. Enzymatic activity estimation

The enzymatic activity for sEH was estimated as the ratio of diol: epoxide (i.e. product:substrate) for the omega-6 (n-6) PUFAs (linoleic acid and arachidonic acid isomeric pairs) and the omega-3 (n-3) PUFAs (alpha linolenic acid, eicosapentaenoic acid, and docosahexaenoic acid isomeric pairs).

### 2.6. RNA-sequencing

RNA isolation and sequencing were performed as previously described (10). Briefly, RNA was isolated from 25 to 30 mg liver using a hand-held OMNI tissue homogenizer and the RNeasy Plus Mini kit (QIAGEN, Valencia, CA) according to the manufacturer’s instructions. RNA quality assessment, RNA-seq library production, and sequencing were performed by Novogene Corporation, Inc. (Sacramento, CA). RNA integrity and quantitation were determined with an Agilent Bioanalyzer 2100 system and the RNA Nano 6000 Assay Kit (Agilent Technologies, Inc., Santa Clara, CA). Sequencing libraries were generated and purified using the NEBNext Ultra RNA Library Prep Kit for Illumina (New England Biolabs, Ipswich, MA), following the manufacturer’s recommendations. Libraries were then sequenced on a NovaSeq 6000 S4 platform (Illumina, Inc., San Diego, CA), generating 150 bp paired-end reads.

Raw reads (FASTQ) were cleaned using fastp (version 0.20.0) and aligned to mouse reference genome GRCm38 with Spliced Transcripts Alignment to a Reference software (version 2.6.1d). Mapped reads were counted for each gene with FeatureCounts (version 1.5.0-p3). edgeR (version 3.22.5) was used for differential expression analysis of two conditions with Benjamini & Hochberg adjusted *p*-values.

### 2.7. Statistical analysis

Statistical analyses were performed using JMP Pro 16 (SAS Institute Inc., Cary, NC; http://www.jmp.com). Outliers were inspected and excluded. Lipids with <20 % missing data were replaced using multivariate normal imputation, otherwise, were excluded. Data normalization was done using Johnson’s transformation and normality was tested using the Shapiro–Wilk test. Least means were calculated after adjusting for liver weight and sex. Non-normalized data were used to calculate lipid geometric means. Fold change (FC) was calculated as A/B where A is the geometric mean of (tx-j or *Atp7b*^−/−^ B6) and B is the geometric mean of (C3H or WT). A FC >1 indicates an increase and <1 indicates decrease, with ±20 % FC set as a threshold.

Student’s *t*-test of Johnson normalized data was used to examine differences between tx-j vs. C3H, and *Atp7b*^−/−^ B6 vs. WT. Mean differences were considered likely at *p* < 0.05. Spearman’s rank correlations were used to examine the correlation between the oxidative stress marker, 9-HETE and other OXLs, performed. To adjust for false discovery rate (FDR), Benjamini-Hochberg FDR correction was performed, with a *q* = 0.1 was set as a threshold [35]. Network/pathway visualizations with fold change and *p*-values were plotted using Cytoscape 3.8.2 (https://cytoscape.org) [36].

### 2.8. Pathway analysis

We integrated lipidomic and transcriptomic data using the joint pathway analysis module from MetaboAnalyst (McGill University, Quebec, CA; http://metaboanalyst.ca). A list of altered OXLs and genes (raw *p* ≤ 0.5) with fold changes were used as input to be compared against species-specific libraries for metabolic pathways containing both lipids and metabolism genes from Kyoto Encyclopedia of Genes and Genomes (KEGG) [37]. Topology analysis was employed to evaluate the importance of a node based on its position within a pathway. For topology analysis and to evaluate the importance of a lipid based on its position within a pathway, the relative betweenness approach was used. Hypergeometric test was used to test the enrichment of lipids related to a particular pathway compared to random hits. All *p*-values are adjusted for multiple testing using Benjamini-Hochberg false discovery rate (FDR) adjustment with *q* = 0.1 [35].

## 3. Results

### 3.1. Both mouse models of WD show traits compatible with liver histopathological changes

**Fig. 1.**
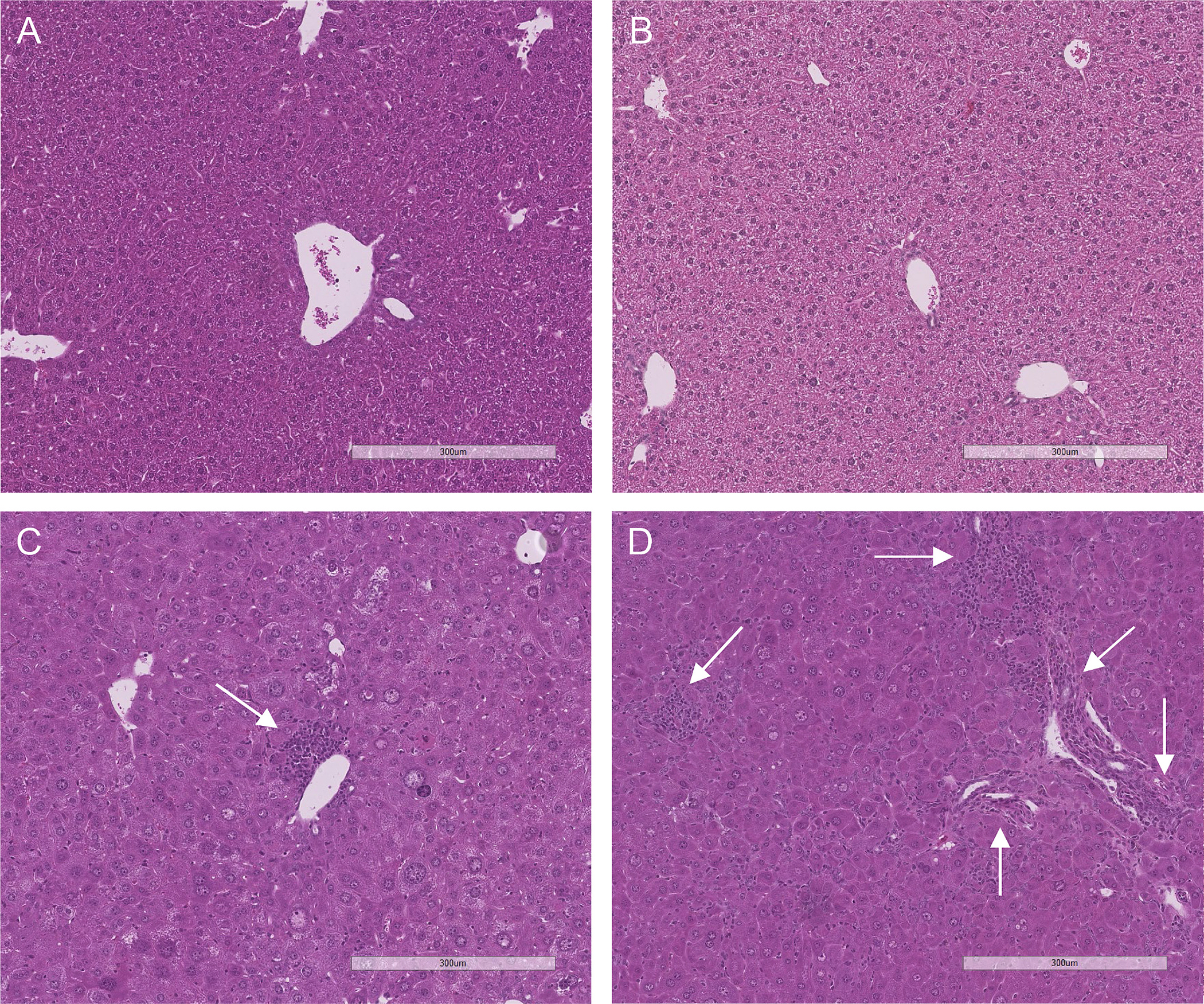
Hematoxylin and eosin staining of representative mouse livers at 16 weeks of age. 10× magnification. Scale bar = 300 μm at 10×. Liver histology is normal in control C3H (1A) and WT (1B) mice. Tx-j (2A) and *Atp7b*^−/−^ B6 (2B) mice presented diffusely enlarged and glycogenated hepatocyte nuclei and both portal and lobular lymphocytic infiltrates (arrows). *Atp7b*^−/−^ B6, The *Atp7b* global knockout on a C57Bl/6 background; C3H, C3HeB/FeJ control mice; tx-j, The toxic milk mice from The Jackson Laboratory; WT, *Atp7b*^+/+^ controls.

In both tx-j and *Atp7b*^−/−^ B6, body and mesenteric white adipose tissue weights were lower compared to corresponding control mice, however, significant only in tx-j mice, with mesenteric white adipose tissue weights about half that of C3H. Liver weight showed opposite trends between models; liver weight was lower in tx-j compared with C3H, whereas in *Atp7b*^−/−^ B6, it was slightly higher than WT. However, compared with respective controls with normal copper metabolism, both WD genotypes showed higher liver weight to body weight ratio, suggestive of liver pathological changes (Table 1). This was further confirmed by histological examination of inflammatory foci, which showed the *Atp7b* mutation and copper accumulation resulted in hepatocellular inflammation with evidence of scattered portal and lobular inflammatory foci in both WD genotypes (Fig. 1). Although we did not attempt direct inter-strain comparisons, *Atp7b*^−/−^ B6 presented more advanced inflammation compared with WT, relative to what was observed in tx-j compared with C3H. Interestingly, when examining control groups, 68 % of WT and 33 % of C3H showed about presence of 1–4 inflammatory foci, indicating strain-related hepatic pathological changes (Table 2). Both tx-j and *Atp7b*^−/−^ B6 had no micro/macrovascular steatosis or signs of fibrosis.

### 3.2. Oxidative stress and auto-oxidation marker is significantly higher in tx-j mice

**Fig. 2.**
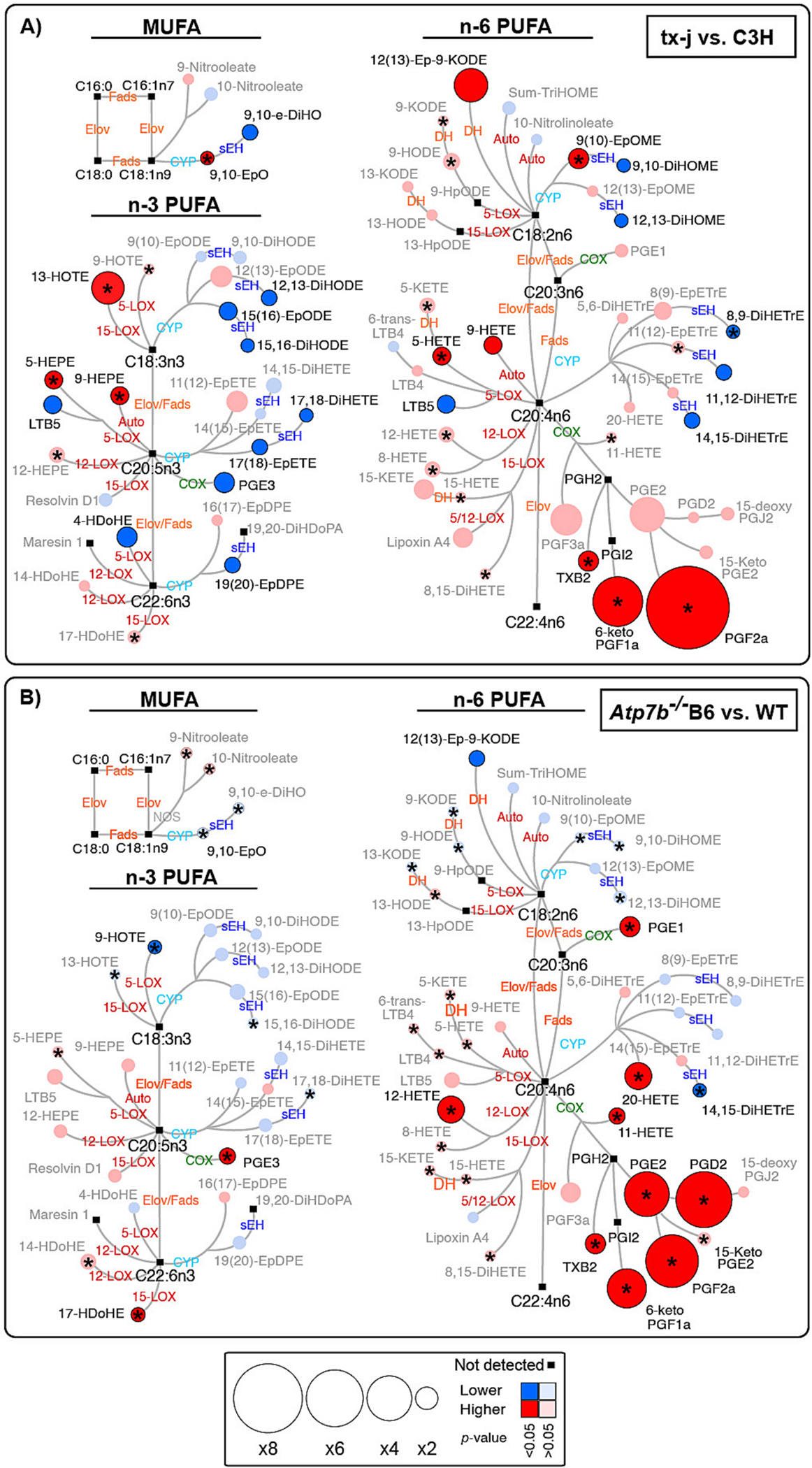
Differences in hepatic oxylipin profiles in two disease mouse models of Wilson’s disease compared with controls. Metabolic network for (A) tx-j vs. C3H and (B) *Atp7b*^−/−^ B6 vs. WT illustrating metabolic pathways for monounsaturated (MUFAs), n-3 and n-6 polyunsaturated fatty acids (PUFAs), and for oxylipin biosynthesis. MUFAs and PUFAs are described by number of carbons and double bonds (i.e. C18:2n6). Nodes size represents fold changes, calculated as A/B where A is the geometric mean of (tx-j or *Atp7b*^−/−^ B6) and B is the geometric mean of (C3H or WT). A fold change <1 indicates an increase and <1 indicates decrease. Means and *p*-values are detailed in (Table S2). Color indicates direction of change – increase in WD (red), decrease in WD (blue), and lipids not detected (black). Color intensity indicates *p*-values – dark (*p* < 0.05 and FDR-adjusted *p* < 0.1) and light (*p* ≥ 0.05 or FDR-adjusted *p* ≥ 0.1). (*) denotes lipids correlating with oxidative stress marker 9-HETE (*r* ≥ 0.4 and <0.6, *p* ≤ 0.05, FDR-adjusted *p* ≤ 0.1). C3H *n* = 12 and tx-j *n* = 18; WT *n* = 25 and *Atp7b*^−/−^ B6 *n* = 23. *Atp7b*^−/−^ B6, The *Atp7b* global knockout on a C57Bl/6 background; Auto, non-enzymatic auto-oxidation; C3H, C3HeB/FeJ control mice; COX, cyclooxygenase; CYP, cytochrome P450 monooxygenase; DH, ehydrogenase; DiHDoPA, dihydroxydocosapentaenoic acid; DiHDPE, dihydroxydocosapentaenoic acid; DiHETE, dihydroxyeicosatetraenoic acid; DiHETrE, dihydroxyeicosatrienoic acid; DiHO, dihydroxyoctadecanoic acid; DiHODE, dihydroxyoctadecadienoic acid; DiHOME, dihydroxyoctadecenoic acid; Elov, fatty acid elongase; Ep-KODE, epoxyoxooctadecenoic acid; EpETE, epoxyeicosatetraenoic acid; EpETrE, EpO, Epoxystearic acid; Epoxyeicosatrienoic acid; EpODE, epoxyoctadecadienoic acid; EpOME, epoxyoctadecenoic acid; Fads, fatty acid desaturase; HDoHE, hydroxydocosahexaenoic acid; HEPE, hydroxyeicosapentaenoic acid; HETE, hydroxyeicosatetraenoic acid; HODE, hydroxyoctadecadienoic acid; HOTE, hydroxyoctadecatrienoic acid; HpODE, hydroperoxyoctadecadienoic acid; KETE, keto-eicosatetraenoic; KODE, keto-octadecadienoic acid; LOX, lipoxygenase; PGE, prostaglandin E; PGF, prostaglandin F; sEH, soluble epoxide hydrolase; TriHOME, trihydroxyoctadecaenoic acid; TXB2, thromboxane B2; tx-j, The toxic milk mice from The Jackson Laboratory; WT, *Atp7b*^+/+^ controls.

To assess the extent of hepatic oxidative stress in early stages of liver disease in WD, we examined the hepatic lipidome in tx-j and *Atp7b*^−/−^ B6 for changes in the arachidonic acid-derived marker of auto-oxidation, 9-HETE. Compared with corresponding control mice, 9-HETE was higher in both genotypes. However, this elevation was significant in tx-j mice (*p* = 0.004) and showed a trend in *Atp7b*^−/−^ B6 mice (Figs. 2, S1 and Table S2). We also observed similar findings for the eicosapentaenoic acid-derived hydroxy fatty acid, 9-HEPE, which was higher in tx-j mice (*p* = 0.002) and showed a similar trend in *Atp7b*^−/−^ B6 mice compared with controls.

Since auto-oxidation can produce mid-chain alcohols and PG-like mediators or isoprostane [20,38–41], we examined the correlations of 9-HETE with these OXLs. Several OXLs correlated positively with 9-HETE in tx-j mice (*r* < 0.4–0.8, *p* ≤ 0.05, FDR-adjusted *p* ≤ 0.1), and in *Atp7b*^−/−^ B6 mice (*r* < 0.4–0.8, *p* ≤ 0.05, FDR-adjusted *p* ≤ 0.1) (Table S3). Correlated OXLs are discussed in relevance to their enzymatic pathways in the following sections. In tx-j mice, a moderate negative association was found between 9-HETE and body, liver, and mesenteric white adipose tissue weights (*r* ≥ 0.4 and <0.8, FDR-adjusted *p* ≤ 0.1). Significant correlations between similar parameters were not observed in *Atp7b*^−/−^ B6 mice.

### 3.3. Higher hepatic thromboxane and prostaglandin levels characterize mice with Wilson disease

Compared with respective controls, both tx-j and *Atp7b*^−/−^ B6 showed a trend for higher PGs and TXBs produced via COX pathway(s). In tx-j mice, this trend was significant for the arachidonic acid-derived TXB_2_ and PGs including, PGF_2_alpha and 6-keto-PGF_1_alpha. The eicosapentaenoic acid derived PGE_3_ was lower. We identified moderate positive correlations between 9-HETE and TXB_2_, PGF_2_alpha and 6-keto-PGF_1_alpha (*r* ≥ 0.4 and <0.8, FDR-adjusted *p* ≤ 0.1) (Fig. 2 and Table S2).

In *Atp7b*^−/−^ B6 mice, a significantly higher level was found for TXB_2_ and many PGs as the arachidonic acid-derived PGF_2_alpha, 6-keto-PGF_1_alpha, PGD_2_, PGE_1_ and PGE_2_; the dihomo-gamma-linolenic acid- and eicosapentaenoic acid-derived PGE_1_ and PGE_3_, respectively. The arachidonic acid-derived alcohol, 11-HETE, that is derived via COX was also higher (Fig. 2 and Table S2). Moderate positive correlations were found between 9-HETE and TXB_2_ as well as many PGs including PGE_1_, PGE_2_, PGD_2_, 6-keto-PGF_1_alpha, and PGF_2_alpha (*r* ≥ 0.4 and <0.8, *p* ≤ 0.05, FDR-adjusted *p* ≤ 0.1) (Table S3). These findings suggest upregulated COX pathways are associated with copper overload in both animal models of WD. The moderate positive correlations of 9-HETE with TXB_2_, and many PGs, in both tx-j and *Atp7b*^−/−^ B6 suggests ROS implications in the origin of these OXLs.

### 3.4. Mice with hepatic copper accumulation show higher PUFA alcohol, ketone, and diol levels

Compared with their respective controls, both tx-j and *Atp7b*^−/−^ B6 showed a trend for higher hepatic PUFA alcohols, ketones, and diols. In tx-j mice, this trend was significant for the arachidonic acid-alcohol 5-HETE, alpha linolenic acid-alcohol 13-HOTE, and eicosapentaenoic acid-alcohol 5- and 9-HEPE. However, we observed lower arachidonic acid-diol LTB_5_ and docosahexaenoic acid-alcohol 4-HDoHE levels (Fig. 2 and Table S2). We found strong positive correlations between 9-HETE and arachidonic acid-alcohols 5- and 8-HETE; eicosapentaenoic acid-alcohol 5- and 9-HEPE (*r* ≥ 0.8, *p* ≤ 0.05, FDR-adjusted *p* ≤ 0.1). There were moderate positive correlations between 9-HETE and many fatty acid alcohols and ketones (*r* ≥ 0.4 and <0.8, *p* ≤ 0.05, FDR-adjusted *p* ≤ 0.1) (Table S3).

In *Atp7b*^−/−^ B6 mice, we identified higher levels of the arachidonic acid-alcohol 12-HETE and the docosahexaenoic acid-alcohol 17-HDoHE, with lower level of the alpha linolenic acid-alcohol 9-HOTE. Strong positive correlations were found between 9-HETE and arachidonic acid-alcohol 8-HETE (*r* ≥ 0.8, *p* ≤ 0.05, FDR-adjusted *p* ≤ 0.1). Moderate positive correlations were found between 9-HETE and many fatty acid alcohols, ketones and diols, including 5-, 8-,12-,15-HETE and 9-HOTE (*r* ≥ 0.4 and <0.8, *p* ≤ 0.05, FDR-adjusted *p* ≤ 0.1) (Table S3). Together, these findings suggest upregulated LOX pathway(s). The correlations observed between 9-HETE and many fatty acid alcohols, ketones and diols in both genotypes suggest a contribution of non-enzymatic auto-oxidation as an origin for these OXLs.

### 3.5. WD mouse models are associated with altered hepatic PUFA epoxides and lower vicinal diols

Compared to their respective controls, both tx-j and *Atp7b*^−/−^ B6 showed altered CYP450-generated PUFA epoxides. However, in tx-j mice, results were more marked with significantly higher levels of the linoleic acid-epoxide 9(10)-EpOME and the epoxy-ketone 12(13)Ep-9-KODE, and in the levels of the oleic acid-epoxide 9,10-EpO (Fig. 2 and Table S2). We observed a trend for lower levels of n-3 PUFA epoxides that were significant for 15,16-EpODE, 17(18)-EpETE, and 19(20)-EpDPE. Also, we observed lower levels of many vicinal diols that was significant for the linolenic acid-derived 9,10- and 12_13-DiHOME; arachidonic acid-derived 8,9-, 11,12-, and 14,15-DiHETrE; alpha linolenic acid-derived 12,13- and 15,16-DiHODE; eicosapentaenoic acid-derived 17,18-DiHETE; and oleic acid-derived 9,10-e-DiHO. We observed some significant changes in the sEH indices in tx-j mice, with a decrease in some enzymatic activities estimated by linoleic acid, arachidonic acid, and alpha linolenic acid isomeric pairs; and an increase in enzymatic activities estimated by eicosapentaenoic acid and docosahexaenoic acid isomeric pairs (Fig. S2 and Table S2). We observed moderate positive correlations between 9-HETE and fatty acid epoxides including 9(10)-EpOME and 9,10-EpO and 11,12-EpETrE; and with the vicinal diols 5,6- and 8,9-DiHETrE (r ≥ 0.4 and <0.6, *p* ≤ 0.05, FDR-adjusted *p* ≤ 0.1). We also observed moderate positive associations between 9-HETE and some sEH activity indices (r ≥ 0.4 and <0.6, *p* ≤ 0.05, FDR-adjusted *p* ≤ 0.1) (Table S3).

In *Atp7b*^−/−^ B6 mice, changes in fatty acid epoxides and diols were less marked. The CYP-derived arachidonic acid-alcohol, 20-HETE, was higher. We observed a trend for lower vicinal diols that was significant for the arachidonic acid-derived 14,15-DiHETrE (Fig. 2 and Table S2). Less marked changes were observed for the estimated sEH activity indices with only decreased activity when estimated by 12_13-DiHOME/12(13)-EpOME (Fig. S2 and Table S2). Moderate positive correlations were found between 9-HETE and 20-HETE; the fatty acid epoxides 9,10-EpO and 9(10)-EpOME; and many vicinal diols (*r* ≥ 0.4 and <0.8, *p* ≤ 0.05, FDR-adjusted *p* ≤ 0.1) (Fig. 2 and Table S2). Also, there were some weak positive associations between 9-HETE and sEH activities estimated (*r* = 0.3, *p* ≤ 0.05, FDR-adjusted *p* ≤ 0.1) (Table S3). These findings suggest altered CYP450 monooxygenase activities in both animal models, which are more marked in tx-j mice compared with C3H control.

### 3.6. Hepatic expression levels of genes related to PUFA metabolism, oxidative stress, and inflammation are altered in WD mouse models

**Fig. 3.**
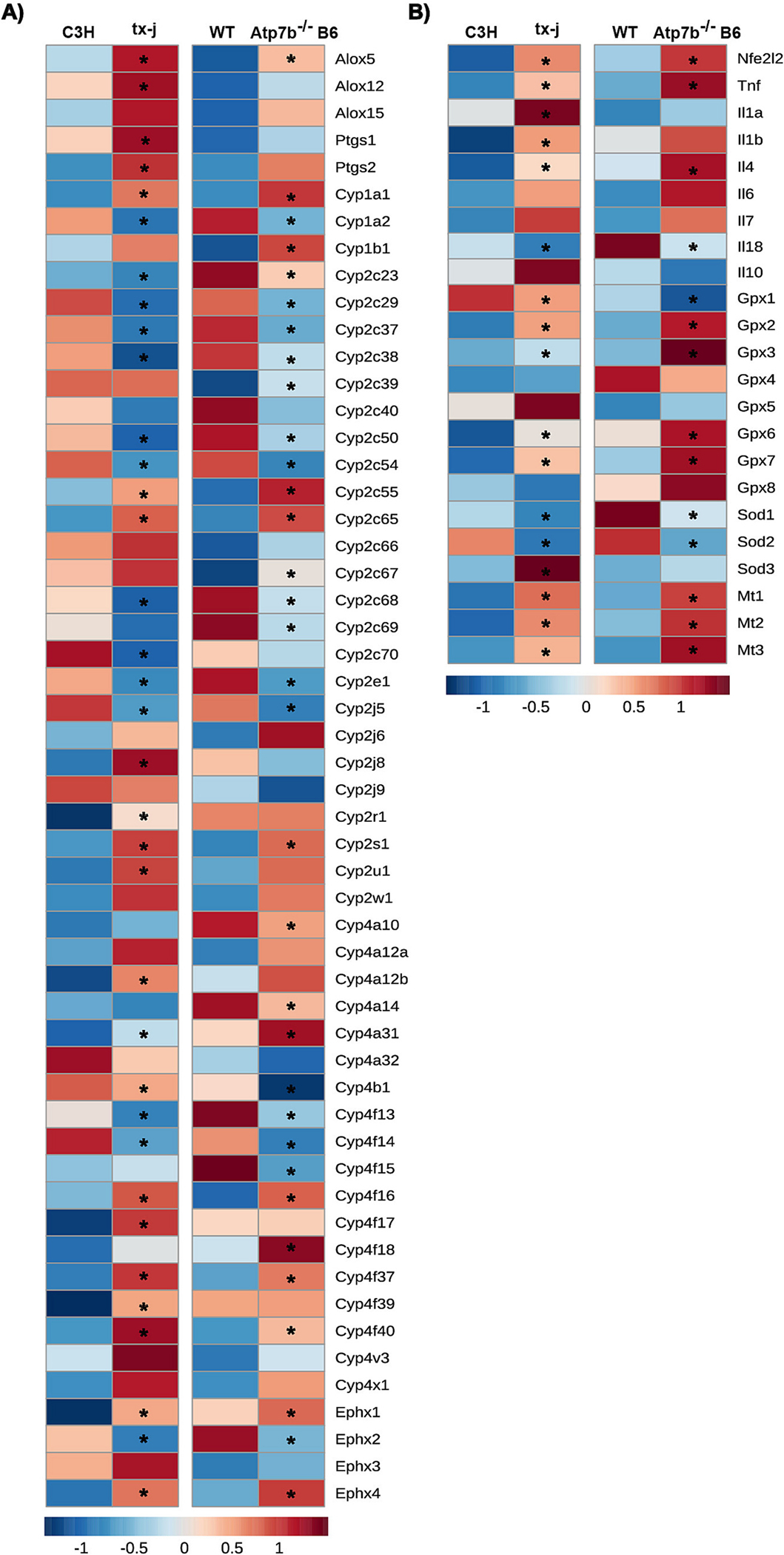
Differences in hepatic gene expression related to PUFA oxidation, oxidative stress and inflammation in two mouse models of Wilson disease compared with controls. Hepatic RNA-seq data of 16-week-old tx-j vs. C3H and *Atp7b*^−/−^ B6 vs. WT mice illustrating (A) genes related to PUFA enzymatic oxidation, (B) genes related to oxidative stress and inflammation. C3H *n* = 6 and tx-j *n* = 6; WT *n* = 6 and *Atp7b*^−/−^ B6 *n* = 6. Data are represented as means ± SEM and statistical significance, (*) indicate *P* < 0.05. *Alox*, Arachidonate lipoxygenase gene; *Atp7b*^−/−^ B6, The *Atp7b* global knockout on a C57Bl/6 background; C3H, C3HeB/FeJ control mice; *Cyp*, Cytochrome P450 monooxygenase gene; *Gpx*, Glutathione peroxidase gene, *Il*, Interleukin genes; *Mt*, Metallothionein gene; *Nfe2l2*, Nuclear factor erythroid 2-related factor2 gene; *Ptgs*, Prostaglandin-endoperoxide synthase gene; PUFAs, Polyunsaturated fatty acids; *Sod*, Superoxide dismutase gene; *Ephx*, Epoxide hydrolases gene; tx-j, The toxic milk mice from The Jackson Laboratory; *Tnf*, Tumor necrosis factor gene; WD, Wilson disease; WT, *Atp7b*^+/+^ controls.

We examined the transcript levels of genes related to enzymatic PUFA oxidation, oxidative stress, and inflammation. Compared to respective controls, hepatic transcript levels in tx-j mice showed LOX genes *Alox5* and *Alox12* significantly upregulated as well as *Ptgs1* and *Ptgs2*, which encode COX-1 and COX-2, and many CYP- and sEH-related genes were significantly dysregulated. Also, the transcript levels of genes related to oxidative stress and inflammation were upregulated, including *Tnf*, *Nfe2l2*, *Il1a*, *Il1b*, Il4, *Gpx1*, *Gpx2*, *Gpx3*, *Gpx4*, *Gpx5*, *Mt1*, *Mt2*, *Mt3*, and *Sod3* (Fig. 3 and Table S4).

*Atp7b*^−/−^ B6 mice showed significantly upregulated hepatic transcript levels of *Alox5*, trends for higher *Ptgs1* and *Ptgs2*, and dysregulation in many CYP- and sEH-related genes. Transcript levels of genes related to oxidative stress and inflammation were upregulated, including *Tnf*, *Nfe2l2*, *Il4*, *Gpx2*, *Gpx3*, *Gpx6*, *Gpx7*, *Mt1*, *Mt2*, and *Mt3*.

### 3.7. Arachidonic acid and linoleic acid metabolism are altered in WD mouse models

The integration of lipidomic and gene expression into metabolic pathway analysis indicates altered PUFA metabolism in animal models of WD. Results from pathway analysis show that the hepatic metabolism of arachidonic acid and linoleic acid are significantly altered in both tx-j and *Atp7b*^−/−^ B6 mice, compared to respective controls (*p* ≤ 0.05, and FDR-adjusted *p* ≤ 0.1) (Fig. S3 and Table S5). Other pathways were also dysregulated in both animal models of WD including retinol metabolism, steroid hormone metabolism, and glutathione metabolism (*p* ≤ 0.05, and FDR-adjusted *p* ≤ 0.1) (Fig. S3 and Table S5).

## 4. Discussion

The current work explored hepatic lipidomic profiling in two animal models of WD, tx-j and *Atp7b*^−/−^ B6 mice. Our findings highlight strain-related predisposition to hepatic inflammation, and indicate that, at 16 weeks of age, mice with early stage WD but with evident hepatic inflammation are characterized by altered PUFA metabolism. The main results include 1) elevated levels of 9-HETE, the ROS-dependent non-enzymatic marker of oxidative stress, that is more marked in tx-j mice, with an upregulation of genes related to oxidative stress and inflammation in both genotypes; 2) higher PGs and TXB, indicating upregulated COX pathways; 3) higher PUFA ketones, diols, and alcohols along with higher transcript levels of *Alox5* indicating upregulated LOX (s) pathways; and 4) altered PUFA epoxides and vicinal diols with altered expression of CYP- and sEH-related genes, indicating dysregulated CYP(s) and sEH(s) pathways.

The genetic loss of function in ATP7B results in copper accumulation, primarily affecting the liver and manifesting with hepatocellular steatosis, necro inflammation, and fibrosis [3]. At 16 weeks of age, both animal models show marked hepatic copper accumulation, with ~60-fold increase in tx-j compared to C3H control mice and ~30-fold in *Atp7b*^−/−^ B6 compared to WT [8,42]. At this time point, liver pathology is still in the early development phases, with evidence of hepatocellular portal and lobular inflammation, however, no steatosis or fibrosis. Our findings are in line with previous observations and are indicative of hepatocellular inflammation that is more evident in *Atp7b*^−/−^ B6 mice [8]. Interestingly, control mice with normal copper metabolism of both strains presented mild inflammatory infiltrates which could indicate a background predisposition to inflammation that is accentuated by copper accumulation. These results are in agreement with previously described strain differences [7,43], and with the strain-related differences in responses to excessive dietary copper reported in other animals [44].

At early stages of liver disease that are associated with hepatocellular inflammation, both tx-j and *Atp7b*^−/−^ B6 mice were characterized by changes in hepatic lipidomic profile and signs of altered PUFA metabolism. The arachidonic acid alcohol 9-HETE is derived from free-radical mediated non-enzymatic oxidation and is a marker of oxidative stress [20]. In tx-j mice, the higher levels of 9-HETE, and the strong positive correlation found between 9-HETE and several PUFA alcohols is consistent with auto-oxidative processes induced by oxidative stress. Changes in 9-HETE were less marked in *Atp7b*^−/−^ B6 compared with WT, however, the moderately positive association between 9-HETE and many PUFA alcohols may suggests auto-oxidation. Findings from gene expression analysis support lipidomic findings with an upregulation in the transcript levels of many genes related to oxidative stress and inflammation. Together, our data indicate lipid auto-oxidation and signs of oxidative stress are evident at early stages (16 weeks of age) in WD mouse models, with possible genotype-related variations. Oxidative stress plays a critical role in the development of many liver diseases as ROS interact with cellular components to perpetuate oxidative damage, inflammation, apoptosis, and necrosis [45]. The role of oxidative stress in the early development of hepatic inflammation in WD is unclear. However, studies from animal models of copper-hepatotoxicity demonstrated that the chronic exposure to sublethal doses of copper induced hepatic oxidative stress via the overproduction of ROS and diminished antioxidants enzymatic activities; increased lipid peroxidation; induced hepatic apoptosis via mitochondrial apoptotic pathway with the activation of the tumor necrosis factor receptor-1 signaling pathway; and provoked liver inflammation by stimulating the mitogen-activated protein kinases and nuclear factor kappa B signaling pathways [44,46–48]. In vitro studies also show that hepatocytes exposure to copper (100 or 200 μM) increased ROS, lipid peroxidation, and resulted in mitochondrial dysfunction and apoptosis via mitochondrial pathway [44,49,50]. In the current study, hepatic liver content was significantly higher in both mouse models, although we did not assess free hepatic copper levels. We also cannot conclude on the source of oxidative stress at early-stage liver inflammation in WD. While current data indicate that unbound copper-generated free radicals at early stages of WD are unlikely, as excess copper is buffered via protective mechanisms, including mitochondria copper sequestering [11,15], structural and functional mitochondrial dysfunction are reported at early stages of WD [51–54], and are suggested to be the first responder to hepatic copper load [54]. Therefore, mitochondrial dysfunction may be a potential sources of ROS at early stages of WD [55].

Our results show both mice models of WD were characterized by higher hepatic levels of the arachidonic acid derived TXB_2_ and several PGs. TXB_2_ and several PGs are produced exclusively via COX pathways (COX-1 and COX-2) and mediate inflammation and necrosis [21,56]. Our findings from gene expression analysis indicate an upregulation of COX pathways with the higher transcript levels of *Ptgs1* and *Ptgs2* detected in tx-j mice, with a trend observed in *Atp7b*^−/−^ B6 mice. An increase in COX expression was reported in Long-Evans Cinnamon rats, an animal model of WD [57]. COX-1 is expressed ubiquitously in most cell types, while COX-2 plays a role in inflammation, angiogenesis, and cell proliferation in a variety of tissues [23,24]. COX-2 is upregulated with inflammation and liver injury and plays an important role in the activation of hepatic stellate cells and fibrogenesis [58]. ROS and COX-2 are shown to be reciprocally linked, with ROS inducing the expression of COX-2 by multiple mechanisms, including the induction by nuclear factor kappa B and COX contributing to inflammation, ROS production, and lipid oxygenation [59–61]. This could explain the moderately positive correlation between both TXB_2_ and prostaglandins with the oxidative stress marker 9-HETE in both mice models of WD. Although sex differences in PG production during inflammation were reported as higher in males [62], we did not observe sex interaction in the current study (data not shown).

In general, both mouse models of WD showed higher PUFA alcohols, ketones, and diols, which suggest an upregulation of LOX pathway(s). Gene expression analysis results are in agreement, as there was upregulation in the transcript levels of LOX-related genes in both mouse models. However, the positive correlation observed between 9-HETE and some PUFA alcohols in both models also suggests a contribution of non-enzymatic pathways in the origin of these OXLs. Animal studies show LOX pathways play a key role in the pathogenesis of liver disease [63]. In general, n-6 PUFA-derived alcohols are pro-inflammatory [21]. Therefore, the alterations observed in PUFA alcohols may be relevant to early stages of hepatocellular inflammation in WD. Together, our findings indicate upregulation of LOX pathways with contribution of non-enzymatic ROS-dependent oxidation pathway in both WD mouse models.

The CYP450-generated PUFA epoxides were altered in both mice models of WD along with dysregulation in the transcript levels of many CYP genes. We also observed altered PUFA vicinal diols profile with dysregulation in the transcript levels of sEH genes. These findings indicate dysregulated CYP and sEH pathways in both mouse models of WD. In general, PUFA epoxides have anti-inflammatory pro-resolving properties, though with a relatively short half-life as they are radially hydrolyzed to vicinal diols that are inactive or less potent [22]. The implication of these alteration on hepatic inflammation and the observed genotype-related differences awaits further examination.

In agreement with our lipidomic and gene expression findings that indicate altered PUFA metabolism in animal models of WD, results from pathway analysis show that the hepatic metabolism of arachidonic acid and linoleic acid are significantly altered in both tx-j and *Atp7b*^−/−^ B6 mice, compared to controls.

In conclusion, we present data indicating that early stages of liver pathology in WD animals is associated with altered PUFA metabolism. In specific, our findings highlight signs of oxidative stress and non-enzymatic PUFA oxidation that are more evident in tx-j mice. They also indicate enzymatic PUFA oxidation via COX and LOX pathways, with alterations in hepatic arachidonic acid and linoleic acid metabolism in both tx-j and *Atp7b*^−/−^ B6 mice. We postulate a role of these pathways in early stages of WD that needs to be further examined and confirmed in humans. Our findings support antioxidant therapy to alleviate oxidative stress and suggest COX and LOX inhibitors as potential therapeutic targets. Our results also imply the utility of high n-3 PUFA supplementation, which can modulate inflammation by conversion to anti-inflammatory lipid mediators [64,65]. Of note, in the Long-Evans Cinnamon rat, a mixture of docosahexaenoic acid and soy supplementation resulted in improved liver histological outcomes and downregulation in the expression of COX-2 [57].

Our findings further highlight genotype-related variations. The underlying cause of this variability has yet to be elucidated, but it is reflective of the varied human WD phenotypes and it is well described in different mouse strains [66]. Our data show that at 16 weeks of age, compared to correspondent controls, both mouse models showed increased oxidative stress that was more marked in tx-j mice, upregulated COX and LOX, with altered CYP and sEH(s) levels. However, *Atp7b*^−/−^ B6 mice were characterized by more advanced inflammatory infiltrate (>4 foci). Interestingly, despite the advanced liver pathology, this model demonstrates remarkable hepatic regeneration [7], which is indicated by the observed higher liver weight as compared to body weight. These findings imply the existence of protective mechanisms against copper overload and suggest the *Atp7b*^−/−^ mouse model as a candidate for studying compensatory mechanisms in liver disease related to copper toxicity [7].

## Supplementary Material

Supplementary Figures

Supplementary Tables

## Figures and Tables

**Table 1:** Body weight and composition for mice with WD at 16 weeks of age.

*n* (F/M)	C3H	tx-j	FC	*p*-Value	WT	*Atp7b*^−/−^ B6	FC	*p*-Value
	12 (6/6)	18 (10/8)			25 (13/12)	23 (11/12)		

BWt (g)	31.30 ± 2.19	24.70 ± 2.28	0.79	1.70E-07	26.40 ± 3.27	25.40 ± 2.78	0.96	0.364
LWt (g)	1.68 ± 0.15	1.41 ± 0.07	0.84	5.20E-08	1.20 ± 0.36	1.38 ± 0.20	1.15	0.0511
MWAT (g)	0.275 ± 0.100	0.133 ± 0.039	0.484	2.0E-06	0.113 ± 0.057	0.091 ± 0.023	0.805	0.161
LWt:BWt	0.054 ± 0.003	0.057 ± 0.004	1.070	0.00964	0.045 ± 0.011	0.054 ± 0.008	1.200	0.00032
LCu* (μg/g)	4.84 ± 0.17	260.1 ± 8.78	53.70	3.2E-10	7.42 ± 1.25	194.8 ± 12.35	26.26	5.14E-11

Data are presented as mean ± SEM (nominal data). Comparisons were performed by t-test.

* n (F/M) for liver copper: C3H 10 (5/5), tx-j 10 (5/5), WT 17 (12/5), KO 17 (11/6).

*Atp7b*^−/−^, *Atp7b* global knockout on a C57Bl/6 background; BWt, Body weight; C3H, C3HeB/FeJ control for tx-j; FC, Fold change; LCu, Liver copper; LWt, Liver weight; MWAT, Mesenteric white adipose tissue; tx-j, The Jackson Laboratory toxic milk mouse; WT, *Atp7b*^*+/+*^ wild type control for *Atp7b*^−/−^.

**Table 2:** Liver histological characteristics for mice with WD at 16 weeks of age.

*n* (F/M)		C3H	tx-j	*p*-Value	WT	*Atp7b*^−/−^B6	*p*-Value
		12 (6/6)	18 (10/8)		25 (13/12)	23 (11/12)	

	0 foci	8 (67)	1 (6)	0.0005	8 (32)	0 (0)	0.0033
Inflammation	1–2 foci	3 (25)	9 (50)	0.178	12 (48)	5 (22)	0.0628
	3–4 foci	1 (8)	7 (38)	0.0712	4 (16)	2 (9)	0.471
	>4 foci	0 (0)	1 (6)	0.733	1 (4)	16 (69)	0.0001

Data are presented as percent (categorical data). Comparisons were performed by chi-square test (categorical).

*Atp7b*^−/−^B6, *Atp7b* global knockout on a C57Bl/6 background; C3H, C3HeB/FeJ control for tx-j; tx-j, The Jackson Laboratory toxic milk mouse; WT, *Atp7b*^+/+^ wild type control for *Atp7b*^−/−^ B6.

## Data Availability

Details of analysis protocols and data reported are available at the NIH Common Fund’s National Metabolomics Data Repository (NMDR) website, the Metabolomics Workbench (http://www.metabolomicsworkbench.org), study ID number (ST002800). The data can be accessed directly via its Project DOI: https://doi.org/10.21228/M8WH9J. All sequencing data have been deposited in NCBI’s BioSample database with BioProject ID PRJNA1046086.

## Abbreviations:

Alox: Arachidonate lipoxygenase gene
*Atp7b*^−/−^ B6: The *Atp7b* global knockout on a C57Bl/6 background
C3H: C3HeB/FeJ control mice
COX: Cyclooxygenase
CYP: Cytochrome P450 monooxygenase
*Cyp*: Cytochrome P450 monooxygenase gene
DiHDoPA: Dihydroxydocosapentaenoic acid
DiHETE: Dihydroxyeicosatetraenoic acid
DiHETrE: Dihydroxyeicosatrienoic acid
DiHO: Dihydroxyoctadecanoic acid
DiHO: Dihydroxy-stearic acid
DiHODE: Dihydroxyoctadecadienoic acid
DiHOME: Dihydroxyoctadecenoic acid
Ep-KODE: Epoxyoxooctadecenoic acid
EpDPE: Epoxydocosapentaenoic acid
EpETE: Epoxyeicosatetraenoic acid
EpO: Epoxystearic acid
EpODE: Epoxyoctadecadienoic acid
FC: Fold change
FDR: false discovery rate
*Gpx*: Glutathione Peroxidase gene
HDoHE: Hydroxydocosahexaenoic acid
HEPE: Hydroxyeicosapentaenoic acid
HETE: Hydroxyeicosatetraenoic acid
HODE: Hydroxyoctadecadienoic acid
HOTE: Hydroxyoctadecatrienoic acid
HpODE: Hydroperoxyoctadecadienoic acid
*Il*: Interleukin genes
KETE: Keto-eicosatetraenoic
KODE: Keto-octadecadienoic acid
LOX: Lipoxygenase
LTB5: Leukotriene B5
*Mt*: Metallothionein gene
*Nfe2l2*: Nuclear factor erythroid 2-related factor2 gen
n-3: Omega-3
n-6: Omega-6
OXLs: Oxylipins
PGE: Prostaglandin E
PGF: Prostaglandin F
PGs: Prostaglandins
*Ptgs*: Prostaglandin-endoperoxide synthase gene
PUFAs: Polyunsaturated fatty acids
ROS: Reactive oxygen species
sEH: Soluble epoxide hydrolase
*Sod*: Superoxide dismutase gene
*Ephx*: Epoxide hydrolases gene
tx-j: The toxic milk mice from The Jackson Laboratory
*Tnf*: Tumor necrosis factor gene
TXB_2_: Thromboxane B_2_
WD: Wilson disease
WT: *Atp7b*^+/+^ controls
