# Supplementary material for: Hepatic oxylipin profiles in mouse models of Wilson disease: New insights into early hepatic manifestations
Source: Biochim Biophys Acta Mol Cell Biol Lipids. Author manuscript; Available in PMC 2024 Jul 5. (PMC11224028; doi:10.1016/j.bbalip.2023.159446)
Supplement: Supplementary Figures [file NIHMS2002469-supplement-Supplementary_Figures.docx]

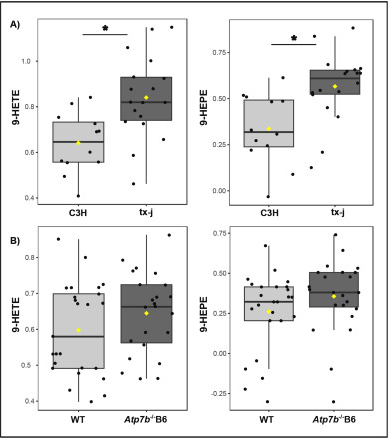


**Fig. S1**. Levels of fatty acids alcohols produced via non-enzymatic pathways in (A) tx-j vs. C3H and (B) Atp7b−/−B6 vs. WT. (*) denotes (p < 0.05 and FDR-adjusted p < 0.1). C3H n = 12 and tx-j n = 18; WT n = 25 and Atp7b−/−B6 n = 23. Atp7b−/−B6, The Atp7b global knockout on a C57Bl/6 background; C3H, C3HeB/FeJ control mice; HEPE, hydroxyeicosapentaenoic acid; HETE, hydroxyeicosatetraenoic acid; tx-j, The toxic milk mice from The Jackson Laboratory; WT, Atp7b+/+ controls.


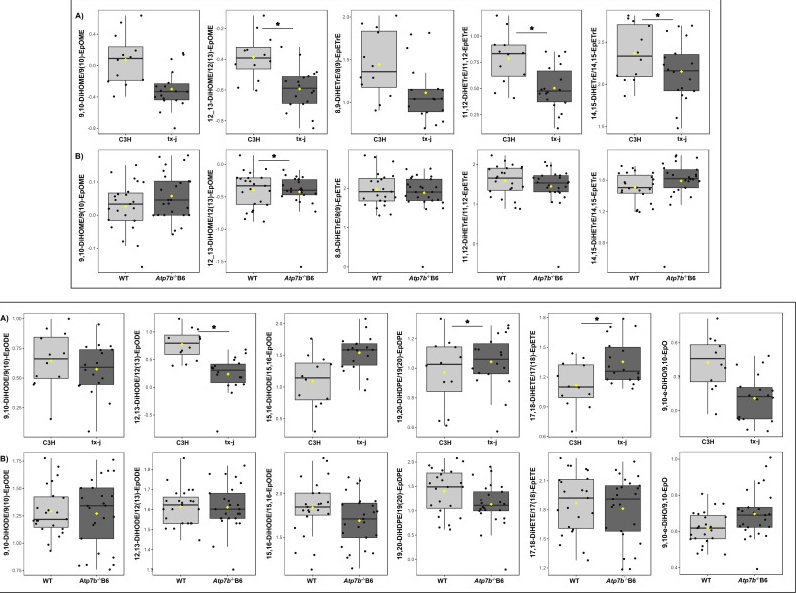


**Fig. S2**. The enzymatic activity for soluble epoxide hydrolase (sEH) in (A) tx-j vs. C3H and (B) Atp7b−/−B6 vs. WT. The sEH activity is estimated as the ratio of diol:epoxide for the n-6 PUFAs isomeric pairs, linoleic acid and arachidonic acid isomeric pairs (top panel); the n-3 PUFAs, alpha linolenic acid, eicosapentaenoic acid, and docosahexaenoic, and oleic acid isomeric pairs (top panel). (*) denotes (p < 0.05 and FDR-adjusted p < 0.1). C3H n = 12 and tx-j n = 18; WT n = 25 and Atp7b−/−B6 n = 23. Atp7b−/−B6, The Atp7b global knockout on a C57Bl/6 background; C3H, C3HeB/FeJ control mice; DiHDoPA, dihydroxydocosapentaenoic acid; DiHDPE, dihydroxydocosapentaenoic acid; DiHETE, dihydroxyeicosatetraenoic acid; DiHETrE, dihydroxyeicosatrienoic acid; DiHO, dihydroxyoctadecanoic acid; DiHODE, dihydroxyoctadecadienoic acid; DiHOME, dihydroxyoctadecenoic acid; EpETE, epoxyeicosatetraenoic acid; EpETrE, Epoxyeicosatrienoic acid; EpO, Epoxystearic acid; EpODE, epoxyoctadecadienoic acid; EpOME, epoxyoctadecenoic acid; tx-j, The toxic milk mice from The Jackson Laboratory; WT, Atp7b+/+ controls.


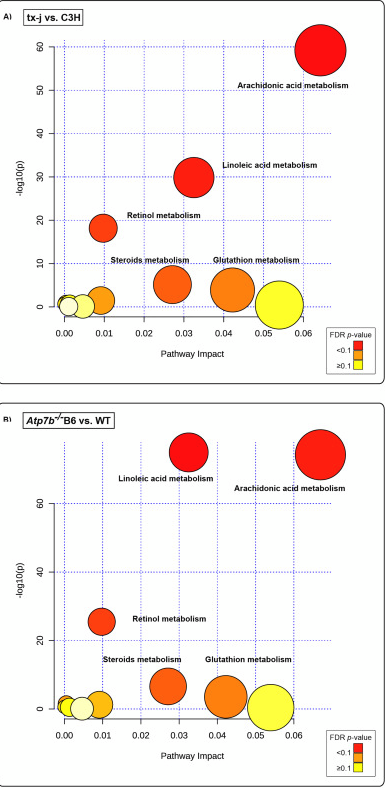


**Fig. S3**. Alteration in hepatic OXL pathways in mouse models of Wilson disease. Results for integrated metabolic pathway analysis for (A) tx-j vs. C3H and (B) Atp7b−/−B6 vs. WT. Lipids altered in (raw p < 0.2) were compared against library from Kyoto Encyclopedia of Genes and Genomes (KEGG). Metabolic pathways significantly altered are shown as nodes. The (y-axis) represents the significance (p-values) determined by Fisher's Exact test. The (x-axis) illustrate the pathway impact assessed by the relative betweenness centrality-topology analysis. The size of the node represents the total hit number of hits. Detailed pathway analysis statistics are shown in (Table S4).
